# Real-world evaluation of cinacalcet on hard outcomes in hemodialysis patients in Saudi Arabia

**DOI:** 10.1186/s12882-025-04455-y

**Published:** 2025-09-26

**Authors:** Riad Mohammed Abdelrahman, Azmi Mohamed Ali Abdallah, Ayaz Ahmed Ahmed, Taha Hussien Musa, Ismail Adam Arbab, Eltieb Omer Abuelgasim, Mohammed Jalal, Kannan O. Ahmed, Yousif Amin Hassan, Zainab Altrefe, Huda Babikir Ahmed

**Affiliations:** 1 Faculty of Graduate Studies and Scientific Research, National Ribat University, Khartoum, Sudan; 2 Faculty of Pharmacy, National Ribat University, Khartoum, Sudan; 3 Nephrology Unit, Al-Mana General Hospitals, Al-Khobar, Saudi Arabia; 4 Pharmacy Department, Mohammed Al Mana College for Medical Sciences, AL Khobar, Saudi Arabia; 5 Pharmacy Program, School of Medical & Health Sciences, Libyan International Medical University, Benghazi, Libya; 6 School of Medicine & Biomedical Research Institute, Darfur University College, Nyala, Sudan; 7 Faculty of Pharmacy, Department of Pharmacy Practice, International University of Africa, Khartoum, Sudan; 8 Pharmacy College, Karary University, Khartoum, Sudan; 9 Department of Pharmacy Practice, College of Pharmacy, National University of Science and Technology, Muscat, Sultanate of Oman; 10 Associate Professor, Department of Clinical Pharmacy and Pharmacy Practice, Faculty of Pharmacy, University of Gezira, Wad Medani, Sudan

**Keywords:** Secondary hyperparathyroidism (SHPT), Chronic kidney disease (CKD), iPTH, Cinacalcet

## Abstract

**Background:**

Chronic Kidney Disease (CKD) complications, like cardiovascular complications, are one of the leading causes of mortality. Managing the biochemical profile is essential in slowing the progression of CKD and its associated consequences. This study aimed to assess the effect of cinacalcet add-on therapy on clinical outcomes in patients with End-Stage Renal Disease (ESRD) on Hemodialysis (HD) who developed Secondary Hyperparathyroidism and are receiving hemodialysis.

**Method:**

A mixed retrospective-prospective cohort multicenter study was conducted in three hospitals in Saudi Arabia between December 1st, 2019, and January 31st, 2021.

**Results:**

One hundred and seventy-four subjects were analyzed; the incidence of the cardiovascular events and the first cardiovascular events in patients on cinacalcet was significantly decreased compared to the patients on the conventional therapy (*p* = 0.02 & 0.005,* respectively*). The incidence of coronary artery diseases was significantly decreased by 61% in the patients on cinacalcet (*HR = 0.39*, *p* = 0.04). Patients on Cinacalcet were 69% less in all-cause first hospitalization hazard ratio; *HR* = *0.31*,* 95% CI: 0.16–0.63*, *p* = 0.001. There was no significant reduction in the risk of all-cause and cardiovascular mortality in the patients on Cinacalcet (*p* = 0.06 & 0.12,* respectively*), but patients with Hypertension and “Diabetes Mellitus & Hypertension” etiology had a lower mortality *HR* (Hypertension: *HR = 0.46*,* 95% CI = 0.21-1.00*, *p* = 0.05; “Diabetes Mellitus & Hypertension”: *HR* = *0.42*,* 95% CI = 0.2-1.00*, *p* = 0.04). There was no significant difference in the frequency of incidence of bone fractures between the two groups (*p* = 0.26).

**Conclusion:**

Cinacalcet is superior in decreasing the frequency of cardiovascular events. However, it is not effective in reducing the risk of all-cause and cardiovascular mortality, except in patients with “Hypertension” and “Diabetes Mellitus & Hypertension” etiology, and it might offer a somewhat protective trend in males and older patients.

**Clinical trial number:**

Not applicable.

**Supplementary Information:**

The online version contains supplementary material available at 10.1186/s12882-025-04455-y.

## Background

Chronic Kidney Disease (CKD) is defined as abnormalities in kidney structure or function that present for three months or longer [1]. It is associated with serious health implications, including an increased risk of kidney failure, cardiovascular disease, reduced quality of life, and premature mortality. Early identification and management are therefore crucial to slowing disease progression and mitigating complications [1].

A new classification system was recommended by the Kidney Disease Improving Global Outcomes (KDIGO), which is called the Cause, Estimated Glomerular Filtration Rate (GFR), and Albuminuria (CGA) Staging System, that incorporated the GFR and urine albumin-creatinine ratio [2].

CKD affects about 10% of the overall population worldwide, and diabetes and high blood pressure are the most common causes of the disease [3]. In recent decades, chronic kidney disease (CKD) has emerged as a significant health concern in Saudi Arabia, largely because of the increasing incidence and prevalence of end-stage renal disease (ESRD) among its population. An epidemiological study reported that the overall prevalence of CKD in the country was 4.76% [4].

Chronic Kidney Disease–Mineral Bone Disorder (CKD-MBD) is a complex syndrome, defined by the KDIGO as a term used to collectively describe the mineral (e.g., phosphorus, calcium, parathyroid hormone (PTH)), bone (osteodystrophy), and soft-tissue calcification that develop as a complication of CKD. Mineral bone disorders represent a clinical condition that, when present and not sufficiently controlled, forecasts a very high risk of death and Cardiovascular (CV) events [3].

Secondary hyperparathyroidism (SHPT) is a common complication of CKD and a component of CKD-MBD, arising from hyperphosphatemia, hypocalcemia, and the balance among hyperparathyroidism, reduced production of active vitamin D, and resistance to vitamin D [5].

Management of CKD generally based on KDIGO clinical guidelines.

Pharmacologic management of PTH, phosphorus, and calcium balance is essential in preventing the development of SHPT and slowing CKD and associated consequences [2, 6].

KDIGO guidelines suggested an intact Parathyroid Hormone (iPTH) goal of 2–9 times the upper limit of normal for patients with SHPT-CKD on dialysis (which corresponds to a range of 130–600 pg. /mL) [7]. Control of iPTH levels with Vitamin D receptor activators- which include Calcitriol, Doxercalciferol, Paricalcitol, and Alfacalcidol-is associated with improved overall survival in chronic kidney disease (CKD) patients [8, 9].

In patients with CKD stage 5 requiring PTH-lowering therapy, KDIGO suggests calcimimetics, calcitriol, or Vitamin D analogs or a combination of calcimimetics with calcitriol or Vitamin D analogs [10].

According to many studies, Cinacalcet effectively reduces PTH levels and the biochemical profile of patients with SHPT-CKD [11–13].

Achieving these therapeutic targets remains challenging due to the inherent limitations of conventional therapies. Emerging evidence indicates that several drug classes confer renoprotective effects beyond those provided by standard treatment modalities. Sodium-glucose cotransporter 2 (SGLT2) inhibitors have shown efficacy not only in patients with diabetic kidney disease but also in those with non-diabetic CKD, demonstrating significant reductions in renal and cardiovascular outcomes [14].

Similarly, incretin-based therapies, particularly GLP-1 receptor agonists, have been reported to improve kidney outcomes in diabetic populations, with growing evidence supporting their broader protective role [15]. A recent narrative review further summarized that the mainstay treatments of Diabetic kidney disease are the renin–angiotensin system (RAS) inhibitors, SGLT2 inhibitors, incretin-based therapeutic agents, and mineralocorticoid receptor antagonists (MRA) [16].

However, the introduction of cinacalcet had a positive impact on the biochemical profile and morbidity. However, no RCT has definitively shown the Cinacalcet reduces mortality of patients with SHPT-CKD [17, 18].

Data collected and analyzed in 2010 in the United States found a significant survival benefit associated with a cinacalcet prescription for intravenous Vitamin D patients [19].

A meta-analysis found that calcimimetics treatment effectively improved the biochemical parameters of patients with SHPT receiving dialysis without increasing all-cause mortality and all adverse events [20].

A heated debate arose following the publication of the Evaluation of Cinacalcet Hydrochloride Therapy to Lower Cardiovascular Events (EVOLVE) trial in 2012, which failed to demonstrate cinacalcet clinical usefulness. Imbalance in subjects’ baseline levels of PTH, kidney function, and age at randomization and discontinuation rates had been suggested as reasons for the lack of mortality benefit. When these covariates were accounted for in post hoc analyses, the impact of the imbalance became apparent, showing that cinacalcet significantly improved clinical outcomes in patients with CKD, with the exception of mortality, where no clear effect was observed [21].

Given the inconsistency of these findings, it has been suggested that cinacalcet should not be recommended for reducing cardiovascular events or improving survival [22]. 

Post-EVOLVE trial, the investigation of cinacalcet’s influence on clinical outcomes became a focus, and numerous studies have since reported negative conclusions [23–25].

However, many studies later have emphasized the positive role of cinacalcet in controlling the biochemical as well as the clinical consequences of SHPT in patients with CKD on dialysis [26–32].

Many clinical trials and observational studies have been conducted in Saudi Arabia to explore the effect of cinacalcet on the biochemical profile of patients with SHPT [33–35].

One of these studies has concluded that cinacalcet has a beneficial effect on suppressing iPTH and achieving control over bone biochemistry [36]. However, no study has comprehensively investigated the clinical endpoints of cinacalcet use in patients with SHPT.

Despite the advent of cinacalcet and non-calcium-containing phosphate binders, controlling the progression of Vascular Calcification is still challenging.

Our study offers valuable insight into the effects of cinacalcet in the Saudi Arabian population, addressing a gap in randomized controlled trial evidence, and provides data that may inform both local practice and the broader international nephrology community.

## Methods

### Study type

This mixed retrospective and prospective cohort multicenter study reviewed the electronic medical records (EMRs) of patients with CKD and secondary hyperparathyroidism (SHPT) who were on hemodialysis. The study was initially conducted retrospectively; however, because the number of patients during this period alone was insufficient to reach the target sample size, a prospective phase was added, resulting in a mixed retrospective-prospective design to ensure adequate statistical power.

### Study area

Data were collected from three dialysis centers situated in the Eastern Province of the Kingdom of Saudi Arabia.

### Duration of the study

Retrospective phase: December 1st, 2019, to June 30th, 2020. Prospective phase: July 1st, 2020, to January 31st, 2021.

### Study population

Participants aged ≥ 18 years treated with maintenance hemodialysis thrice per week for ≥ 3 months before screening were eligible.

### Sample size

Cochran’s sample size formula was used, and the sample size was calculated based on the estimated prevalence of CKD in Saudi Arabia; the sample size was calculated to be 138 [37].

### Inclusion criteria & exclusion criteria

#### Inclusion criteria

1. Patients aged ≥ 18 years treated with maintenance hemodialysis thrice weekly for ≥ 3 months before screening.
2. Participants receive Cinacalcet and/or conventional therapy (consisting of Alfacalcidol: 0.05–0.1 mcg/kg/day + Sevelamer: 800 mg TID).
3. Plasma iPTH levels of 300 pg. /mL or higher, serum-corrected total calcium levels of 8.4 mg/dL or less than 10.2 mg/dL, or signs of severe SHPT as determined by nephrologists at the dialysis centers.

### Treatment

The treatment assignment was according to the local guidelines in the nephrology unit at Al-Mana Hospitals. Medications assigned to patients are shown in the table below;

### Medications assigned to patients

**Table:** 

Cinacalcet add-on group	Conventional therapy alone group*
Cinacalcet (Mimpara^®^) 30 mg once daily (titrated as needed)	
Alfacalcidol (One alpha^®^ PO or IV); 0,05-0.1 mcg/kg/day	Alfacalcidol (One alpha^®^ PO or IV); 0,05-0.1 mcg/kg/day
Phosphate binder (Sevelmer) (Renvella^®^ 800 mg ×3).	Phosphate binder (Sevelmer)(Renvella^®^ 800 mg ×3).
Calcium Carbonate (Seacal^®^)	Calcium Carbonate (Seacal^®^)
Recombinant erythropoietin IV (Darbepoetin alfa (Aranesp^®^)); SC doses of 0.45 mcg/kg administered once per week and 0.75 mcg/kg once every other week.	Recombinant erythropoietin IV (Darbepoetin alfa (Aranesp^®^)); SC doses of 0.45 mcg/kg administered once per week and 0.75 mcg/kg once every other week.
Ferrosac^®^ (Iron sucrose) IV, 100 mg (5 mL) during 10 consecutive HD sessions to provide the total dose of 1 g.	Ferrosac^®^ (Iron sucrose) IV, 100 mg (5 mL) during 10 consecutive HD sessions to provide the total dose of 1 g.

^*^Conventional therapy includes dietary modification to reduce phosphate intake. All patients were on HD treatment three times per week (4 h/session)

#### Exclusion criteria

1. Parathyroidectomy in the 12 weeks before the start of cinacalcet; history of seizure within 12 weeks.
2. Scheduled for a kidney transplant, the subject is pregnant or breastfeeding, or the subject has known sensitivity or intolerance to any of the protocol-required therapies.

### Data collection

We collected the data from routine sources, dialysis department records, and the hospital network’s nephrologists’ interface.

The following demographic variables were collected: Age, gender, and age ranking (< 65 and ≥ 65 years) were recorded, as well as iPTH levels. We also gathered the following clinical variables: the cause of CKD, the number of cardiovascular events (Myocardial Infarction, Hospitalization for Unstable Angina, Heart Failure, Peripheral Vascular Event), and mortality (all-cause and cause-specific), and fractures.

### Statistical analysis

Data were entered in a Microsoft Excel file. However, the data description and analysis were done using the IBM SPSS statistical software program SPSS (v. 26). A descriptive study was initially carried out, and the data were reported by the mean ± standard deviation, minimum, maximum percentage, median, and percentiles depending on the variable. We used the Mann-Whitney U test for continuous variables and the chi-square test for discrete variables throughout the analysis to test the significance of differences between groups and subgroups. To determine treatment effect modifiers, the following factors were prespecified for subgroup analysis: age, gender, age ranking (< 65 and ≥ 65 years), etiology of CKD, and baseline plasma iPTH. Significance was set as *P* ≤ 0.05.

The data’s normality was assessed, and none of the variables was normally distributed (both Kolmogorov-Smirnov and Shapiro-Wilk were *p* < 0.05). Therefore, continuous variables are reported as medians with interquartile ranges (IQR, 25th and 75th percentiles).

Kaplan-Meier time-to-event curves are used for unadjusted and adjusted cardiovascular event endpoints and mortality analyses. The log-rank (Mantel-Cox) test was used for significance (≤ 0.05). The relative hazard (Cinacalcet add-on versus conventional alone) and 95% CIs were calculated using Cox proportional-hazards regression models to provide a relative event rate between groups and subgroups. The significance of Levene’s Test for Homogeneity of Variance (≤ 0.05) was considered.

## Results

### Patients’ demographic characteristics

At cohort enrollment, 261 patients were included. Eighty-five patients were excluded from the analysis due to incomplete or unavailable laboratory biochemical data, and an additional two patients were excluded during the study following kidney transplantation.174 patients were identified for final analysis: 114 in the conventional therapy group and 60 in the Cinacalcet group.

The median age of the patients was 61 years in the total sample, 60.5 in the cinacalcet group, and 62 years in the conventional therapy group. The male’s age was significantly higher than the female’s (*p* = 0.002). Age distribution (young vs. older) was comparable in the main groups and subgroups (*p* = 0.16). Patients were followed for a median follow-up of 12 months in the total sample. The demographics and clinical characteristics of patients are depicted in Table 1.

**Table 1:** Participant demographics and clinical characteristics (*n* = 174)

Variable	% of patients on Cinacalcet ± SD or median (25th;75th percentile) *n* = 60 (%34.5)	% of patients on Conventional therapy ± SD or median (25th;75th percentile) *n* = 114 (65.5%)	*p*-value
**Age (years)**			
* Median*	*60.5(55.25*,*73.25)*	*(57*,*73)*	*0.50*
* Max*	*90*	*88*	
* Min*	*42*	*42*	
***Age ranking***			*0.16* ^*a*^
* Young*	*(62%) ± 0.5*	*(55%) ± 0.5*	*0.52*
* Older*	*(38%) ± 0.5*	*(45%) ± 0.5*	*0.44*
***Gender***			*0.002* ^*b*^
* Male*s	*(70%) ± 0.46*	*(62%) ± 0.49*	*0.49*
* Females*	*(30%) ± 0.46*	*(38%) ± 0.49*	*0.33*
***Etiology of CKD***			
* HYT*	*0*	*(15%) ± 0.40*	*0.000*
* DM*	*(75%) ± 0.50*	*(45%) ± 0.50*	*0.006*
* HYT + DM*	*(42%) ± 0.5*	*(29%) ± 0.47*	*0.12*
* Unknown*	*0*	*(11%) ± 0.31*	*0.001*
***Baseline Laboratory***			
* Median iPTH (pg./ml)*	*516.00(399.75*,*1027.75)*	*459(368.50*,*703.50)*	*0.01*
* Median* *Calcium(mg/dl)*	8.20(7.73,8.50)	8.60(8.18,9.00)	*0.000*
* Median Phosphorus(mg/dl)*	5.65(4.63,7.00)	4.90(4.28,5.80)	*0.003*
***Hypocalcemia***	*26*	*40*	*0.37*
***Average No of hypocalcemic events***	*2.5*	*0.8*	*0.017*

a; p-value for young Vs older, b; p-value for males Vs females

HYT: Hypertension, BPH: Benign Prostatic Hyperplasia, DM: Diabetes Mellitus, iPTH: intact Parathyroid Hormone

**Fig. 1:**
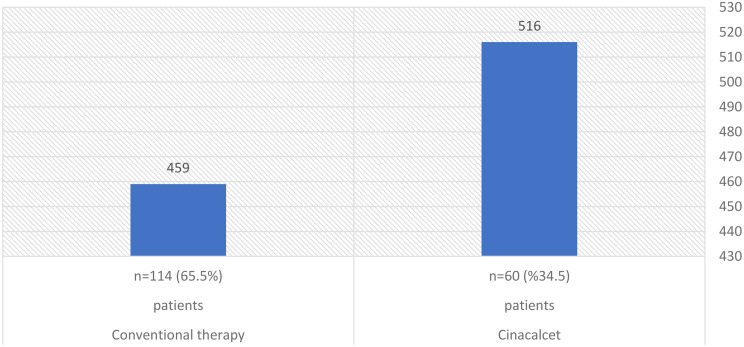
Baseline laboratory: Median PTH (pg./ml)

**Fig. 2:**
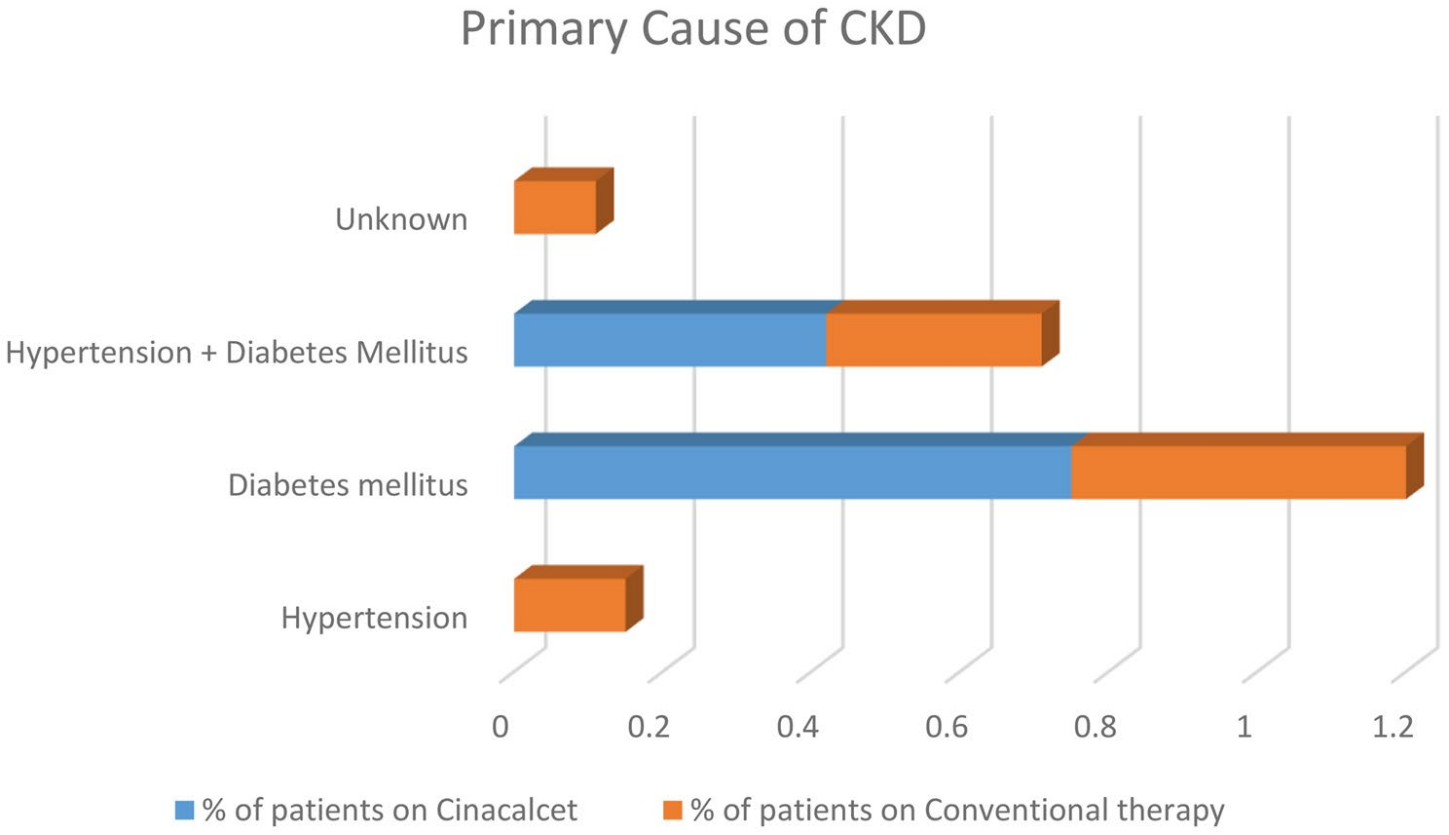
Primary Cause of CKD

Table 1; Figs. 1 and 2 displays the iPTH, calcium, and phosphorus levels and CKD’s etiology.

The etiology of CKD in more than half of the total sample was Diabetes Mellitus (51%), while 33% of patients had CKD caused by both hypertension and diabetes mellitus. Hypertension and unknown primary causes of CKD were significantly more common in the conventional therapy group (*p* = 0.000 & 0.001, respectively). On the other hand, Diabetes Mellitus as a primary cause of CKD was higher in the cinacalcet group (*p* = 0.006).

The baseline median of iPTH and phosphorus was significantly higher in patients on Cinacalcet (p-value 0.01 and 0.003, respectively). Conversely, median serum calcium was significantly higher in the conventional therapy group compared to the Cinacalcet group (p-value 0.000).

The difference between the two groups in the incidence rate of hypocalcemia was not statistically significant (*P* = 0.37). However, there was a significant difference in the average number of hypocalcemic events in the Cinacalcet group compared to the conventional therapy group (p-value 0.017).

### Cardiovascular events

During follow-up, 63 patients (36%) experienced cardiovascular events, most commonly ischemic heart disease or myocardial infarction (*n* = 28; 44%). A total of 53 patients (30%) experienced their first cardiovascular event, primarily due to IHD/MI (*n* = 27; 51%).

The frequency of cardiovascular events-all was significantly lower in patients on cinacalcet compared to patients on conventional therapy (*p* = 0.02) but not in the cardiovascular events specific to a cause.

Table 2 shows that a total of 63 patients (36%) experienced cardiovascular events during follow-up, with ischemic heart disease (IHD) or myocardial infarction (MI) being the most frequent cause of hospitalization (*n* = 28; 44%). Regarding first cardiovascular events since medication initiation, 53 patients (30%) experienced an event, with IHD/MI accounting for the majority (*n* = 27; 51%).

**Fig. 3:**
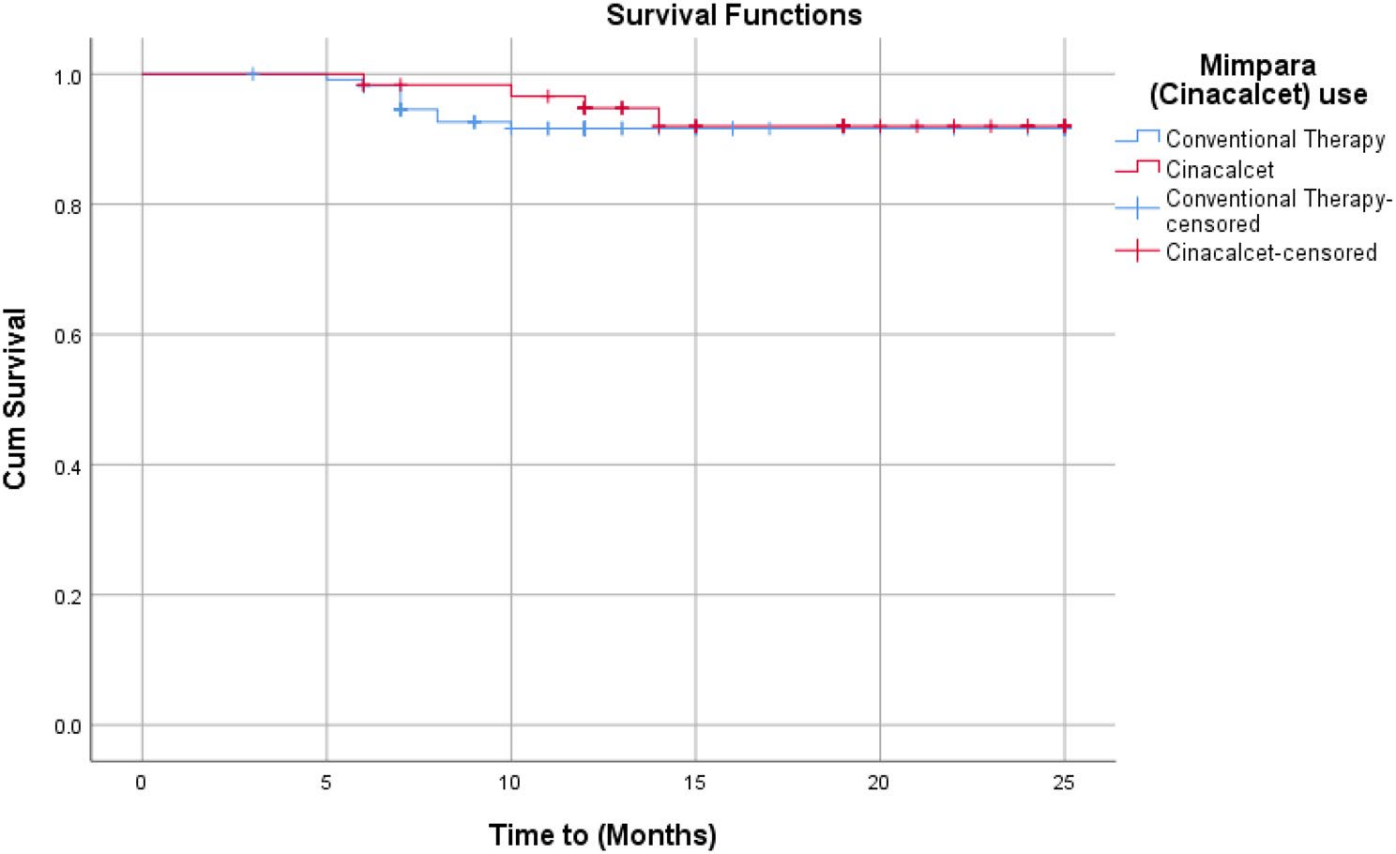
Kaplan-Meier plot for All Cardiovascular events

Patients in the cinacalcet group had a significantly lower frequency of first cardiovascular events compared to the conventional therapy group (*p* = 0.005). For cause-specific first events, a significant reduction was observed only for IHD/unstable angina/MI (*p* = 0.05), while no significance was found for other cardiovascular events. Kaplan-Meier analysis (Table 3; Fig. 3) showed no significant difference between groups for all cardiovascular events (*p* = 0.061); however, the cinacalcet group had a significantly lower rate of IHD/unstable angina/MI hospitalizations (*p* = 0.044).

The Kaplan–Meier curve also indicated that the cinacalcet group experienced their first cardiovascular event later than the conventional group (*p* < 0.001). Cox proportional hazards analysis (Table 5) demonstrated a 69% lower incidence of all-cause first hospitalizations in the cinacalcet group (HR = 0.31, *p* = 0.001). The HR for first IHD/unstable angina/MI hospitalizations was similarly reduced (HR = 0.39, *p* = 0.04). After adjustment for age, age ranking (young vs. older), and gender, the first cardiovascular event risk remained stable across ages, though younger patients on cinacalcet had significantly higher HRs compared to older patients. Males on cinacalcet showed a 33% lower incidence of first hospitalization, representing a non-significant trend toward cardiovascular protection (age: HR = 1.03, *p* = 0.02; age ranking: HR = 2.06, *p* = 0.02; gender: HR = 0.67, *p* = 0.21). No significant differences were observed for other cause-specific cardiovascular events.

### Mortality

**Fig. 4:**
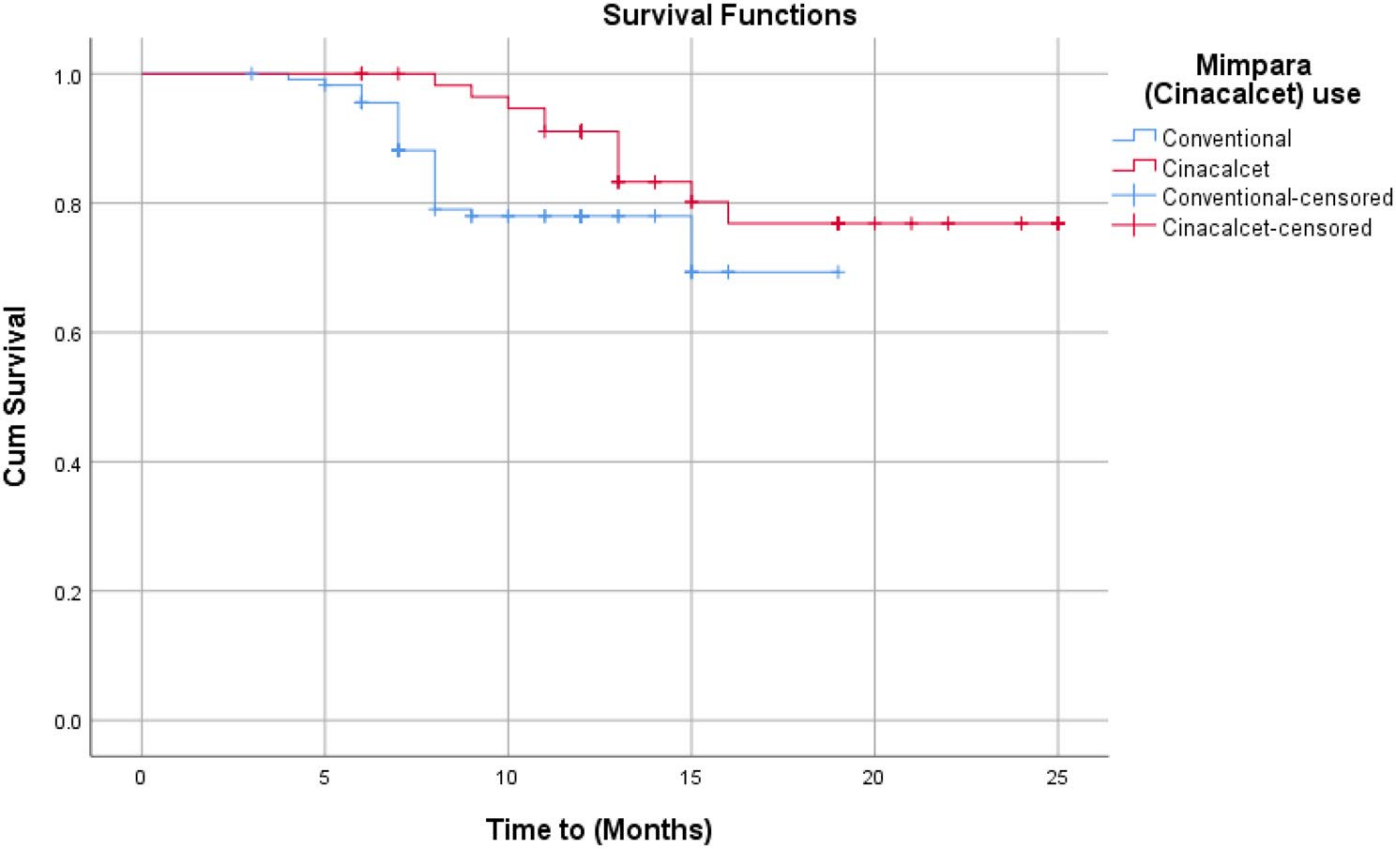
Kaplan-Meier plot for all-cause mortality

All-cause mortality occurred in 34 patients (20%), with cardiovascular disease accounting for 91% of deaths (Table 2). There was no significant difference between the cinacalcet and conventional therapy groups in all-cause mortality (*p* = 0.52) or cardiovascular mortality (*p* = 0.87). Kaplan-Meier analysis (Table 3; Fig. 4) confirmed no significant difference in survival between groups for all-cause (*p* = 0.06) or cardiovascular mortality (*p* = 0.11). Cox proportional hazards analysis (Table 4) also showed no significant reduction in mortality risk with cinacalcet (all-cause: *p* = 0.06; CV: *p* = 0.12).

After adjusting for age, gender, age ranking, baseline PTH, and primary cause of CKD, no survival benefit was observed (all *p* > 0.05). However, patients on cinacalcet with hypertension (HYT) or diabetes mellitus plus hypertension (DM + HYT) as the underlying etiology showed a marginally significant reduction in hazard ratios (*p* = 0.05 and 0.04, respectively). Cardiovascular mortality did not differ significantly after adjustment.

### Bone fractures

No significant differences were observed between the cinacalcet and conventional therapy groups in the frequency of bone fractures (*p* = 0.26, Table 2). Kaplan-Meier analysis also showed no significant benefit for cinacalcet, both unadjusted (*p* = 0.15) and adjusted for gender (*p* = 0.15) or age ranking (*p* = 0.2, Table 3). The unadjusted hazard ratio for fracture was higher in the cinacalcet group (HR = 4.855, *p* = 0.2, Tables 4 and 5), and no effect modification was observed for age, gender, or age ranking (*p* = 0.045, 0.2, and 0.7, respectively).

**Table 2:** Clinical outcomes: frequency of clinical outcomes (*n* = 174)

Clinical outcomes		Cinacalcet No, %± SD (*n* = 60)	Conventional No, %± SD (*n* = 114)	*P*-value Chi-square
*Cardiovascular events*	*Cardiovascular events-all*	*14(23%) ± 0.35*	*49(42%) ± 0.65*	*0.02*
	*IHD/ Hospitalization for Unstable Angina/ MI*	*6(10%) ± 0.34*	*22(19%) ± 0.66*	*0.09*
	*HF*	*1(2%) ± 0.22*	*9(7%) ± 0.77*	*0.096*
	*CVD*	*4(7%) ± 0.50*	*9(7%) ± 0.50*	*0.80*
	*AF& flutter*	*1(2%) ± 0.40*	*3(3%) ± 0.60*	*0.65*
	*P.V. D*	*2(3%) ± 0.38*	*6(5%) ± 0.63*	*0.48*
*First Cardiovascular events*	*First Cardiovascular events*	*10*	*43*	*0.005*
	*First IHD/ Hospitalization for Unstable Angina/ MI*	*6*	*21*	*0.05*
	*First HF*	*1*	*7*	*0.16*
	*First CVD*	*1*	*7*	*0.16*
	*First Atrial fibrillation& flutter*	*1*	*3*	*0.08*
	*First P.V. D*	*1*	*5*	*0.414*
*Mortality*	*All-cause mortality* *CV mortality*	*10 (16.67%)* *10 (16.67%)*	*24 (21.05%)* *21 (18.42%)*	*0.52* *0.87*
*Bone Fractures*		*1*	*6*	*0.26*

IHD: Ischemic Heart Disease, HF: Heart Failure, AF: Atrial Fibrillation, MI: Myocardial Infarction, CVD: Cerebrovascular disease, P.V.D: Peripheral Vascular Disease, CV: Cardiovascular

**Table 3:** Clinical outcomes: Kaplan-Meier analysis (*n* = 174)

Variable		No of patients on Cinacalcet	No of patients on Conventional therapy	Median &95% CI Or Patients No. for Cinacalcet	Median &95% CI Or Patients No. for Conventional therapy	Log Rank (Mantel-Cox)
*IHD Events*		*6*	*22*	*23.276(21.978–24.574)*	*21.275(19.876–22.674)*	*0.044*
*HF Events*		*1*	*9*	*24.764(24.305–25.223)*	*23.505(22.569–24.441)*	*0.084*
*CVD events*		*4*	*9*	*23.881(22.816–24.946)*	*23.516(22.587–24.446)*	*0.606*
*AF events*		*1*	*3*	*24.696(24.107–25.286)*	*24.495(23.932–25.058)*	*0.650*
*PVD Events*		*2*	*6*	*24.508(23.835–25.182)*	*23.977(23.180-24.773)*	*0.483*
*First Cardiovascular events*		*10(17%)*	*43 (38%)*	*22.246(20.698–23.794)*	*17.701(15.992–19.409)*	*0.000*
*All-cause mortality*		*10*	*24*	*22.097 (20.488–23.706)*	*16.052(14.941–17.164*	*0.06*
*Cardiovascular mortality*		*10*	*21*	*22.097 (20.488–23.706)*	*16.312(15.209–17.416)*	*0.11*
*Bone fracture unadjusted*		*1*	*6*			*0.15*
*Bone fracture adjusted for Gender*	*Males*	*1*	*2*			*0.15*
	*Females*	*0*	*4*			
*Bone fracture adjusted for Age Ranking*	*Young*	*0*	*1*			*0.2*
	*Older*	*1*	*5*			

IHD: Ischemic Heart Disease, HF: Heart Failure, CVD: Cerebrovascular disease, AF: Atrial Fibrillation, P.V.D: Peripheral Vascular Disease

**Table 4:** Clinical outcomes: Cox proportional analysis (*n* = 174) (Conventional therapy as reference)

Variable	*Cinacalcet* (*n* = 60)	*Conventional* (*n* = 114)		Cox proportional analysis		
				Hazard Ratio	95% Confidence Interval	*P*-value
***First Cardiovascular events***	*10*	*43*		*0.31*	*(0.16 to 0.63)*	*0.001*
*First IHD/ Hospitalization for Unstable Angina/ MI*	*6*	*21*		*0.39*	*(0.16 to 0.97)*	*0.04*
*First HF*	*1*	*7*		*0.22*	*(0.03 to 1.72)*	*0.15*
*First CVD*	*1*	*7*		*0.8*	*(0.24 to 2.68)*	*0.71*
*First AF& flutter*	*1*	*3*		*0.6*	*(0.062-5.73)*	*0.65*
*First P.V. D*	*1*	*5*		*0.53*	*(0.11 - 2.64)*	*0.44*
***All-cause mortality***	*10*	*24*	*0.52*	*0.48*	*(0.22 to 1.04)*	*0.06*
***Cardiovascular mortality***	*10*	*21*	*0.87*	*0.53*	*(0.24 to 1.17)*	*0.12*
*HF*	*7*	*15*	*0.84*			
*CVD*	*1*	*5*	*0.76*			
*IHD/MI*	*2*	*1*	*0.32*			

IHD: Ischemic Heart Disease, HF: Heart Failure, MI: Myocardial Infarction, CVD: Cerebrovascular stroke, AF: Atrial Fibrillation, P.V.D: Peripheral Vascular Disease

**Table 5:** Clinical outcomes: Cox regression model on time to event (covariates-adjustment) (*n* = 174)

Variable	Hazard Ratio (95% CI)	*P*-value
**Cardiovascular events:** (**All-cause and cause-specific**)		
***All-cause***	*0.31 (0.16 to 0.63)*	*0.001*
*Age*	*1.03 (1.01 to 1.06)*	*0.02*
*Gender (ref*,* female)*	*0.67 (0.36 to 1.25)*	*0.21*
*Young Vs. Older (ref*,* Young)*	*2.06(1.14 to 3.71)*	*0.02*
*First*,* IHD*	*0.39 (0.16 to 0.97)*	*0.04*
*First HF*	*0.22 (0.03 to 1.72)*	*0.15*
*First CVD*	*0.80 (0.24 to 2.68)*	*0.71*
*First AF*	*0.6(0.062–5.73)*	*0.65*
*First P.V.D*	*0.53 (0.11 to 2.64)*	*0.44*
***Mortality: (All-cause mortality)***		
*Treatment (Conventional Therapy/cinacalcet)*	*0.48 (0.22 to 1.04)*	*0.06*
*Age*	*0.34 (0.11 to 1.04)*	*0.06*
*Gender (ref*,* male)*	*0.48 (0.22 to 1.06)*	*0.07*
*Young Vs. Older (ref*,* Young)*	*0.50 (0.23 to 1.11)*	*0.09*
*Baseline PTH*	*1.73 (0.28–10.55)*	*0.6*
*DM*	*0.60 (0.27–1.29)*	*0.2*
*HYT*	*0.46 (0.21-1.00*	*0.05*
*DM + HYT*	*0.42(0.2- 1.00)*	*0.04*
***Mortality: (Cardiovascular mortality)***		
*Treatment (Conventional Therapy/cinacalcet)*	*0.53 (0.24 to 1.17)*	*0.12*
*Age*	*0.36 (0.11 to 1.10)*	*0.7*
*Gender (ref*,* female)*	*0.53 (0.24 to 1.20)*	*0.12*
*Young Vs. Older (ref*,* Young)*	*0.55 (0.25 to 1.25)*	*0.15*
*Baseline PTH*	*1.73 (0.28–10.55)*	*0.6*
*DM*	*0.68 (0.307–1.51)*	*0.35*
*HYT*	*0.51 (0.23–1.13)*	*0.10*
*DM + HYT*	*0.47 (0.203–1.07)*	*0.07*
***Bone fracture***		
*Treatment (Conventional Therapy/cinacalcet)*	*4.855(0.548–43.035)*	*0.2*
*Age*	*1.146 (1.003–1.309)*	*0.045*
*Gender (ref*,* female)*	*0.22(0.03–2.02)*	*0.2*
*Young Vs. Older (ref*,* Young)*	*0.53 (0.014- 17.838)*	*0.7*

P.V.D: Peripheral Vascular Disease, AF: Atrial Fibrillation, IHD: Ischemic Heart Disease, CVD: Cerebrovascular Disease, DM: Diabetes Mellitus, HYT: Hypertension, PTH: Parathyroid Hormone

## Discussion

### Cardiovascular events

Many randomized controlled trials have analyzed the treatment effects of Cinacalcet on hard clinical outcomes. However, a final conclusion remains elusive.

Elevated serum levels of phosphorus, calcium, PTH, and Fibroblast Growth Factor 23 (FGF-23) have been linked to death and negative cardiovascular outcomes [38, 39].

Our findings are more comprehensive as they offer evidence of crude incidence, survival, and hazards of CV events and the adjustment for many covariates: age, age ranking, gender, iPTH levels, and primary cause of CKD.

In the intermediate and late stages of CKD, non-traditional risk factors accelerate progression of CV disease, and arterial disease in patients with CKD is characterized by an almost unique propensity to calcification and vascular stiffness [40]. Given that all our study participants are in stage 5 CKD, we noticed a high incidence of CV events in patients on conventional therapy; this warrants the need for more aggressive treatment measures to control the underlying causes of cardiovascular complications resulting from electrolyte imbalances and the subsequent SHPT.

Our results revealed a statistically significant reduction in the frequency of the incidence of cardiovascular events in patients on Cinacalcet compared to patients on conventional therapy; this significance was not seen in the cause-specific cardiovascular events. These findings, while encouraging, should be interpreted with caution due to potential limitations such as sample size, residual confounding, and the observational design of the study. Therefore, further well-designed, adequately powered prospective studies or randomized controlled trials are warranted to more definitively assess the role of cinacalcet in reducing both overall and specific cardiovascular events in patients with CKD.

Also, we reported a significant benefit of cinacalcet use in reducing the frequency of the incidence of first cardiovascular events. Regarding the cause-specific first event, significance was evident in the first IHD/hospitalization incidence for unstable angina/MI but not in the rest of the first cause-specific events.

These results are similar to what D.C. Wheeler found in a post hoc analysis 2014. He reported that combining fatal and nonfatal cardiovascular events and randomization to cinacalcet reduced the rates of heart failure. He also added that patients randomized to cinacalcet experienced fewer nonatherosclerotic cardiovascular events, while the effect of cinacalcet on atherosclerotic events did not reach statistical significance [30].

These observations suggest that cinacalcet may confer cardiovascular protection primarily through mechanisms affecting nonatherosclerotic processes, although further studies are needed to clarify its effects on specific cardiovascular subtypes.

Our survival analysis-Kaplan-Meier curve revealed that the cinacalcet group significantly had a first cardiovascular event later compared to the conventional therapy group; these results are in agreement with C. Friedl et al. while disagreeing with Y. Sun et al., who failed to prove the survival advantage of cinacalcet in dialysis patients. The discrepancy between studies may be attributable to differences in study design, patient population characteristics, follow-up duration, or concomitant therapies, highlighting the need for further well-designed investigations to clarify the cardiovascular benefits of cinacalcet in this population [32, 41].

We also reported that patients on cinacalcet have a 69% lower incidence of all-cause first hospitalization. These results are consistent with previously published results [19].

On adjustment for age, age ranking (young vs. older), and gender, the first cardiovascular events do not change concerning age. On adjustment for age ranking, young patients have significantly higher HR than older patients. Although males exhibited a 33% lower incidence of first hospitalization, the difference was not statistically significant, indicating only a trend toward mild protection against cardiovascular-related hospitalizations in males.

Our cause-specific analysis demonstrated that the hazard ratio for first ischemic heart disease–related hospitalization, including unstable angina and myocardial infarction, was significantly lower in the cinacalcet group compared to conventional therapy, corresponding to a 61% reduction in these events. These findings align with Cunningham et al., who, in a combined analysis of four Phase III randomized controlled trials, reported that cinacalcet significantly reduced the risk of cardiovascular hospitalizations while showing a non-significant trend toward reduced all-cause mortality [42].

Similarly, Asada et al. observed a reduction in overall hospitalizations, particularly those associated with cardiovascular events, suggesting a modest protective effect of cinacalcet, although these results did not reach statistical significance [43].

Together, these observations support the potential role of cinacalcet in reducing cardiovascular morbidity in patients with SHPT on hemodialysis, highlighting the importance of considering cardiovascular outcomes when optimizing therapy in this population.

### Mortality

Block et al. described a significant survival benefit associated with Cinacalcet prescription in a prospective observational study. These observations and those of others led to the development of a prospective RCT evaluating the effect of cinacalcet treatment on cardiovascular mortality: the EVOLVE study [19].

The EVOLVE study was an RCT that enrolled 3883 hemodialysis patients with moderate to severe SHPT. The primary composite endpoint was time until death, myocardial infarction, and hospitalization for unstable angina, heart failure, or a peripheral vascular event. Although the primary analysis of the EVOLVE trial was negative, prespecified additional analysis showed a significant reduction of the risk of death or cardiovascular outcomes, which suggests a potential benefit of cinacalcet [19, 44, 45].

In addition, a predetermined protocol analysis by Parfrey P.S. et al. revealed that cinacalcet reduced the risk of death and major cardiovascular events in older but not younger patients (*p* = 0.01)) [46].

Wheeler DC et al. analyzed the effects of cinacalcet on atherosclerotic and nonatherosclerotic cardiovascular events in patients receiving hemodialysis in the EVOLVE study; they found that the relative hazard of cardiovascular death randomization to cinacalcet resulted in a statistically significant reduction in the cardiovascular death rate [30].

Moreover, Friedl C et al. reported a significantly lower all-cause mortality (*p* = 0.001) and cardiovascular mortality (*p* = 0.001) in the cinacalcet group [32].

Conversely, Palmer et al., Sin H.Y. in 2016, and Li Xiaosong et al. have found that cinacalcet had little or no effect on all-cause mortality [24, 25, 29].

Our findings provide a more comprehensive perspective by presenting both unadjusted cardiovascular event data and adjusted analyses that account for multiple covariates, including age, age group, gender, iPTH levels, and primary etiology of CKD. In our cohort, we found no statistically significant difference in the frequency of all-cause or cardiovascular mortality between the cinacalcet and control groups.

Kaplan-Meier analysis revealed that the survival of patients using Cinacalcet is not significantly different from that of conventional therapy patients regarding all-cause mortality. The same test showed no significant difference between Cinacalcet patients and conventional therapy patients regarding mortality resulting from CV diseases.

Our Cox proportional analysis findings showed that treatment with Cinacalcet did not significantly reduce the risk of all-cause mortality and cardiovascular mortality.

All-cause mortality adjustment for age, gender, age ranking (young vs. older), and baseline PTH revealed no significant difference between the two groups, but when adjusting for the primary cause of CKD, patients on cinacalcet with HYT and DM + HYT etiology benefit from cinacalcet because they have lower HR.

Also, CV mortality adjustment for the same covariates revealed no significant difference between the two groups.

### Bone fractures

Patients with CKD have an increased risk of fractures compared with the general population [47, 48].

No RCT has been specifically designed to evaluate whether any compounds used in treating SHPT (phosphate binders, vitamin D analogs, or calcimimetics) decrease the risk of fracture in CKD patients. However, treatment with cinacalcet is associated with a reduced risk of fractures [49].

Moe SM et al. tested the data from the EVOLVE study. They concluded that Cinacalcet reduced the rate of clinical fracture [40].

In our study, there was no significant difference in the frequency of incidence of bone fractures in the two groups. The Kaplan-Meier and Cox proportional analyses revealed no significant difference between the two groups. Also, no effect modification is evident for age, gender, or age ranking. However, for gender and age ranking, although it is not significant, a somewhat protective trend is apparent in males and older patients.

### Study limitations

Randomized controlled trials (RCTs) remain the gold standard for establishing treatment efficacy and safety, particularly in large and heterogeneous patient populations. While observational studies can provide insights into real-world clinical practice, their findings are inherently more susceptible to bias and confounding. Although our study was observational, the data were randomly collected from several Al-Mana Hospital patients, which may allow cautious extrapolation to the broader CKD population; however, these results should be interpreted as complementary to, rather than a replacement for, evidence derived from well-conducted RCTs. Furthermore, since the study enrollment began before cinacalcet was officially added to the Al-Mana Hospital formulary, all patients who received cinacalcet during the study could be clearly identified as new users. This allowed us to define the exact start of treatment for each patient, providing a precise and unambiguous baseline for evaluating the effects of cinacalcet in our analysis.

Limitations include the observational design and short follow-up period (12 months, which is unlikely sufficient to detect substantial changes in vascular calcification and consequent cardiovascular complications).

Also, in the retrospective part, some of our data were derived from a patient registry, which could have caused unmeasured confounding; moreover, in this study, no data were obtained on phosphate restriction in diet, but only the reports mentioned it. Our study is also limited by the lack of data on glycemic markers, dialysis efficiency measures, iPTH stratification by KDIGO targets, and concomitant use of renoprotective drugs, which may have introduced residual confounding.

## Conclusion

This study represents the first comprehensive comparison of a cinacalcet-based regimen versus conventional therapy in patients with secondary hyperparathyroidism (SHPT) on hemodialysis, with systematic adjustment for relevant covariates where applicable. Our findings demonstrate that cinacalcet positively influences several clinical outcomes in this population. Specifically, cinacalcet significantly reduced the overall incidence and delayed the timing of first cardiovascular events, including ischemic heart disease–related hospitalizations for unstable angina and myocardial infarction, although no significant effect was observed for other cause-specific cardiovascular events.

Analysis of subgroups revealed that younger patients exhibited a higher hazard of first cardiovascular events compared to older patients, while males showed a trend toward modest protection against cardiovascular-related hospitalizations. Cinacalcet did not significantly reduce overall mortality, except in patients with hypertension (HYT) or combined diabetes mellitus and hypertension (DM + HYT) etiology, a group representing over 40% of our cohort, suggesting a potential targeted benefit. Additionally, no significant reduction in bone fracture incidence was observed; however, a subtle protective trend was noted in males and older patients.

Overall, these results reinforce and extend previous observational and clinical trial findings and provide evidence to guide therapeutic strategies in CKD patients. Further well-designed studies are warranted to confirm these observations and to explore the differential benefits of cinacalcet across patient subgroups.

## Supplementary Information

Below is the link to the electronic supplementary material.

Supplementary Material 1

## Data Availability

The data underlying this article will be shared on reasonable request to the corresponding author.

## Abbreviations

CKD: Chronic Kidney Disease
CV: Cardiovascular
CKD-MBD: Chronic Kidney Disease- Mineral and bone disorder
DM2: Diabetes Mellitus Type 2
FGF-23: Fibroblast Growth Factor 23
iPTH: Intact Parathyroid hormone
HR: Hazard Ratio
HYT: Hypertension
KSA: Kingdom of Saudi Arabia
KDIGO: Kidney Disease Improving Global Outcomes
MBD: Mineral and bone disorder
SHPT: Secondary Hyperparathyroidism
