# Supplementary material for: Real-world evaluation of cinacalcet on hard outcomes in hemodialysis patients in Saudi Arabia
Source: BMC Nephrol. 2025 Sep 26;26:528. doi: 10.1186/s12882-025-04455-y (PMC12465577; doi:10.1186/s12882-025-04455-y)
Supplement: Supplementary file 1 — Supplementary Material 1 [file 12882_2025_4455_MOESM1_ESM.docx]

Table S1. Published studies related to “cinacalcet use in patients on hemodialysis”

| **Source** | **Title/ purpose** | **Type of article** | **Major findings** |
| --- | --- | --- | --- |
| H. J. Kim et al. (2013) | Cinacalcet lowering of serum fibroblast growth factor-23 concentration may be independent from serum Ca, P, PTH and dose of active vitamin D in  peritoneal dialysis patients | Randomized controlled study | Cinacalcet treatment was independently associated with the reduction of FGF23 |
| S. Fishbane et al (2008) | Cinacalcet HCl and concurrent low- dose vitamin D improves treatment of secondary hyperparathyroidism in  dialysis patients compared with  vitamin D alone: the ACHIEVE study results | Randomized controlled study | Cinacalcet-D versus Flex-D subjects had a >30% reduction in parathyroid hormone (PTH) (P = 0.001) as well as PTH <300 pg./ml (P = 0.006) |
| Al. Mohammed et al. (2022) | The Effectiveness of Alternate-day Cinacalcet Therapy for Secondary Hyperparathyroidism in Noncompliant  Hemodialysis Patients | Prospective multicenter study | Cinacalcet produced significant reductions in iPTH with  intermittent (three times per week) doses and thus was more cost-effective and had better compliance. |
| P. Susantitaphong et al (2019) | “The effectiveness of cinacalcet: a randomized, open label study in  chronic hemodialysis patients with  severe secondary hyperparathyroidism | Randomized open label study | Therefore, cinacalcet can provide salutary effects on CKD-MBD in severe SHPT and might be an initially effective PTH-lowering therapy prior to surgical parathyroidectomy as well as an  alternative treatment in the patients unsuitable for surgery. |
| P. U. Torres et.al. (2006) | “Cinacalcet HCl: a novel treatment for secondary hyperparathyroidism caused by chronic kidney disease” | Review | Effectiveness of cinacalcet on the control of PTH secretion, along with simultaneous reductions in calcium, phosphorus, and calcium-phosphorus product, give this agent an advantage over  traditional therapies in all levels of severity of SHPT. |
| B. D. Reams et al. (2015) | “Dynamics of cinacalcet use and biochemical control in hemodialysis patients: A retrospective New-user  cohort design, | A retrospective New-user cohort design | Substantial and expected declines in laboratory values occurred following cinacalcet initiation. Early discontinuation and reinitiation of cinacalcet were common and may have occurred  for clinical and economic reasons. |
| G. A. Block et al. (2010) | Cinacalcet hydrochloride treatment significantly improves all-cause and  cardiovascular survival in a large cohort of hemodialysis patients | Prospective observational study | A significant survival benefit associated with cinacalcet prescription in patients receiving i.v. vitamin D. |

| Q. Zhang et al. (2012) | “Effects and Safety of Calcimimetics in End Stage Renal Disease Patients with Secondary Hyperparathyroidism: A  Meta-Analysis | Meta-Analysis | Calcimimetic treatment effectively improved biochemical parameters of SHPT patients receiving dialysis without increasing all-cause mortality and all adverse events |
| --- | --- | --- | --- |
| P. S. Parfrey et al. (2016) | “Lessons Learned from EVOLVE for Planning of Future Randomized Trials in  Patients on Dialysis | Review | The benefit of cinacalcet was suggested in the sub-analyses of the EVOLVE study, in which the potential problems of the trial  were taken into account |
| M. Evans et al. (2018) | “Cinacalcet use and the risk of  cardiovascular events, fractures and mortality in chronic kidney disease patients with secondary  hyperparathyroidism | Retrospective observational study | Cinacalcet treatment improves biochemical abnormalities in the wider CKD population, and adds real-world support that  treating SHPT with cinacalcet may have beneficial effects on cardiovascular outcomes. |
| H. Komaba et.al. (2015) | Cinacalcet and Clinical Outcomes in Dialysis | Review | Favorable effects of cinacalcet on bone metabolism and vascular calcification, providing plausibility to support the beneficial  effects of cinacalcet. Definitive evidence is, however, still lacking, and further efforts should be made to establish the optimal role of cinacalcet in the treatment of SHPT. |
| H. Y. Sin et.al. (2017) | Prospective cohort study: Cinacalcet- mediated lowering of PTH level and cardiovascular disease mortality in  younger Korean patients with stage 5 CKD at a Korean secondary hospital | Prospective cohort study | Cinacalcet was not associated with decreases in all-cause  mortality or CVD mortality in younger stage 5 CKD patients with high PTH levels (>600 pg/mL). |
| Li. Xiaosong et al. (2023) | “Cinacalcet use in secondary hyperparathyroidism: a machine learning-based systematic review | Meta-Analysis | Confirmed the effects of Cinacalcet on reducing serum PTH and calcium and improving phosphate.  Found no difference in all-cause mortality, cardiovascular mortality, and parathyroidectomy. Cinacalcet was associated  with an increased risk of hypocalcemia. |
| S. M. Moe et al. (2015) | “Cinacalcet, Fibroblast Growth Factor- 23, and cardiovascular disease in  Hemodialysis: The Evaluation of Cinacalcet HCl Therapy to Lower  Cardiovascular Events (EVOLVE) Trial.,” | Secondary analysis of a randomized clinical trial | Treatment with cinacalcet significantly lowers serum FGF23. Treatment-induced reductions in serum FGF23 are associated with lower rates of cardiovascular death and major  cardiovascular events. |

| G. Lozano-Ortega et al (2018) | “Effects of calcimimetics on long-term outcomes in dialysis patients: literature  review and Bayesian meta-analysis” | Meta-Analysis | Treatment of secondary hyperparathyroidism with calcimimetic therapy may reduce mortality among patients receiving  maintenance dialysis. |
| --- | --- | --- | --- |
| J. Rottembourg et al. (2019) | “Factors associated with parathyroid hormone control in haemodialysis patients with secondary hyperparathyroidism treated with cinacalcet in real-world clinical  practice: Mimosa study,” | A retrospective observational study | 41.1% of HD patients with SHPT treated with cinacalcet-HCl remained with a PTH above the KDIGO recommended target  after 12 months of treatment. Apart from the possibility of non- compliance, the severity of SHPT appears to be a major factor determining the response to cinacalcet-HCl treatment,  reinforcing the importance of treating SHPT at earlier stages. |
| S. C. Palmer et al. (2013) | “Cinacalcet in Patients with Chronic Kidney Disease: A Cumulative Meta- Analysis of Randomized Controlled  Trials | A cumulative meta-analysis | Cinacalcet reduces the need for parathyroidectomy in patients with CKD stage 5D, but does not appear to improve all-cause or cardiovascular mortality |
| D. C. Wheeler et al. (2014) | “Effects of cinacalcet on atherosclerotic and nonatherosclerotic cardiovascular events in patients receiving  hemodialysis: The evaluation of cinacalcet HCL therapy to lower  cardiovascular events (EVOLVE) trial,” | Post hoc analysis | Patients randomized to cinacalcet experienced fewer nonatherosclerotic cardiovascular events (adjusted relative  hazard 0.84, 95% CI 0.74 to 0.96), while the effect of cinacalcet on atherosclerotic events did not reach statistical significance. |
| F. Yuan et.al. (2018) | “Effect of Cinacalcet Combined with Calcitriol on the Clinical Outcome and Bone Metabolism in Patients on  Hemodialysis with Severe Secondary  Hyperparathyroidism,” | Prospective cohort study | Cinacalcet combined with low dose calcitriol can improve high calcium, high phosphorus, and high iPTH in MHD patients with severe SHPT and also improve bone metabolism |
| C. Friedl et al. (2017) | “Mortality in dialysis patients with cinacalcet use: A large observational  registry study. | A large observational registry study. | Patients those with moderate sHPT, younger age and without diabetes benefit from cinacalcet with reduced overall and  cardiovascular mortality. |
| A. Al-Hwiesh et.al. (2012) | “Efficacy of Cinacalcet for the Treatment of Secondary  Hyperparathyroidism in CKD Patients on Peritoneal or Hemodialysis: The Middle-East Experience, | Prospective cohort study | Cinacalcet effectively lowers parathyroid hormone levels in patients receiving dialysis and having  uncontrolled secondary hyperparathyroidism. |
| S. O. Bashir et.al. (2015) | “Tolerance and Efficacy of a Low Dose  of the Calcimimetic Agent Cinacalcet in | Prospective cohort study | Cinacalcet significantly reduce the serum iPTH levels |

|  | Controlling Moderate to Severe  Secondary Hyperparathyroidism in Hemodialysis Patients,” |  |  |
| --- | --- | --- | --- |
| A. A. Alharthi et.al. (2015) | “Cinacalcet in Pediatric and Adolescent Chronic Kidney Disease,” | Prospective cohort study | Highly significant reduction in iPTH and serum alkaline phosphatase levels was detected post-cinacalcet. The serum calcium (Ca), phosphate (P), and Ca × P product were  unaffected. Treatment was well tolerated with no hypophosphatemia, hypocalcemia, |
| Y. Sun et.al. (2020) | “Efficacy and safety of cinacalcet compared with other treatments for secondary hyperparathyroidism in  patients with chronic kidney disease or end-stage renal disease: a meta-  analysis, | Meta-Analysis | Cinacalcet improved the biochemical parameters in CKD patients, but did not improve all-cause mortality and  cardiovascular mortality. Moreover, cinacalcet can cause hypocalcemia |
| G. A. Block et al. (2004) | “Mineral metabolism, mortality, and morbidity in maintenance  hemodialysis,” | Meta-Analysis | Disorders of mineral metabolism are independently associated with mortality and morbidity associated with cardiovascular  disease and fracture in hemodialysis patients. |
| J. Cunningham et.al. (2005) | “Effects of the calcimimetic cinacalcet HCl on cardiovascular disease, fracture, and health-related quality of life in  secondary hyperparathyroidism | A combined analysis | Randomization to cinacalcet led to significant reductions in the risk of parathyroidectomy, fracture, and cardiovascular  hospitalization, along with improvements in self-reported physical function and diminished pain |
| S. Asada et al. (2019) | “Effectiveness of cinacalcet treatment for secondary hyperparathyroidism on hospitalization: Results from the MBD- 5D study, | Post hoc analysis | Cinacalcet initiation in patients on maintenance hemodialysis had no effect on all-cause and cause-specific hospitalizations.  Although the overall association was statistically not significant, cinacalcet may have a protective association on cardiovascular- related hospitalization in all patients and infection-related  hospitalization in patient with low intact parathyroid hormone. |
| L. Pereira et.al. (2018) | “Old and new calcimimetics for treatment of secondary hyperparathyroidism: Impact on  biochemical and relevant clinical outcomes | Review | Cinacalcet has been demonstrated to effectively reduce PTH and improve biochemical control of mineral and bone disorders in  chronic kidney patients. However, a final conclusion on the effect of cinacalcet on hard outcomes remains elusive. |

| P. S. Parfrey et al. (2015) | “The effects of cinacalcet in older and younger patients on hemodialysis: The evaluation of cinacalcet HCL therapy to lower cardiovascular events (EVOLVE)  trial. | Post hoc analysis | Cinacalcet decreased the risk of death and of major CV events in older, but not younger, patients with moderate to severe HPT who were receiving hemodialysis. |
| --- | --- | --- | --- |
| S. M. Moe et al. (2015) | “Effects of cinacalcet on fracture events in patients receiving  hemodialysis: The EVOLVE trial” | Post hoc analysis | Cinacalcet did not reduce the rate of clinical fracture |
